# Serum IL-27 predicts the severity and prognosis in patients with community-acquired pneumonia: a prospective cohort study

**DOI:** 10.7150/ijms.67028

**Published:** 2022-01-01

**Authors:** Zheng Xu, Xin-Ming Wang, Peng Cao, Chen Zhang, Chun-Mei Feng, Ling Zheng, De-Xiang Xu, Lin Fu, Hui Zhao

**Affiliations:** 1 Respiratory and Critical Care Medicine, Second Affiliated Hospital of Anhui Medical University, Hefei, Anhui Province, 230601, China.; 2 Department of Pharmacy, First Affiliated Hospital of Anhui Medical University, Hefei, Anhui Province, 230022, China.; 3 Department of Toxicology, Anhui Medical University, Hefei, Anhui Province, 230032, China.

**Keywords:** Community-acquired pneumonia, IL-27, Diagnosis, Prognosis, Biomarker.

## Abstract

**Background**: The previous studies have revealed that IL-27 was involved in the pathophysiology of pulmonary inflammatory diseases. However, the role of IL-27 in community-acquired pneumonia (CAP) was unclear. The goal of this research was to explore the associations of serum IL-27 with the severity and prognosis among CAP patients through a prospective cohort study.

**Methods**: The whole of 239 healthy population and 239 CAP patients were enrolled. Fasting blood samples were collected. Inflammatory cytokines were detected using enzyme linked immunosorbent assay (ELISA). Demographic characteristics and clinical information were analyzed.

**Results**: Serum IL-27 on admission was significantly risen in CAP patients compared with control subjects. Besides, serum IL-27 was gradually increased in line with CAP severity scores. Correlative analysis suggested that serum IL-27 was associated with blood routine indices, renal function, liver function, myocardial function and inflammatory cytokines. Linear and logistic regression analyses revealed that serum IL-27 was positively correlated with CAP severity scores. Logistic regression analysis demonstrated that serum higher IL-27 on admission elevated the risks of vasoactive agent usage and longer hospital stay during hospitalization among CAP patients.

**Conclusions**: Serum IL-27 is markedly and positively associated with the severity and poor prognosis among CAP patients, indicating that IL-27 may involve in the pathophysiological process of CAP. Serum IL-27 may be used as a biomarker for diagnosis and prognosis in CAP patients.

## Introduction

Community-acquired pneumonia (CAP) is a broad and serious pulmonary infection disease. Numerous microbial pathogens can cause CAP, including bacteria, viruses, fungi and so on 1. Despite continuous improvement of medical method, CAP is still an infectious disease complicated with high mortality and morbidity in all ages worldwide 2. CAP is one of common infectious diseases cause of death in the America with more than 1.5 million adults hospitalized annually 3. The mortality was about 171.1 per 1000 people in Central Asia, Eastern Europe and Central Europe, 130.8 per 1000 cases in Southeast Asia, eastern Asia and Oceania 4, 5. Morbidity and mortality were higher in patients with severe CAP 6. Furthermore, the survivors of CAP patients may suffer from new comorbidities or the development of comorbid condition. CAP is typically diagnosed based on clinical presentation and imaging method. However, laboratory examination and imaging test always have hysteresis characteristics for CAP patients. Hence, it's helpful and significant to seek for an effective biomarker to predict the illness among CAP patients.

Interleukin-27 (IL-27) is a heterodimeric IL-12 family cytokine that can be formed from the IL-12p35-related, p28, and EBI3 subunits 7. Antigen-presenting cells (APCs) are a main source of IL-27 release, which can be elicited in response to infectious agents or the stimulation of Toll-like receptors (TLRs) 8. IL-27 signaling can induce the release of a range of pro-inflammatory cytokines from keratinocytes, mast cells and monocytes 9. IL-27 in T cells is a pro-inflammatory cytokine and ultimately enhances the polarization of CD4^+^ cells towards a Th1 phenotype 10. The previous studies have found that the level of IL-27 is increased in patients with tuberculosis, asthma, influenza, acute lung injury, lung cancer, chronic obstructive pulmonary disease (COPD), acute lung injury (ALI) and acute respiratory distress syndrome (ARDS) 11-15. It has been demonstrated that the level of circulating IL-27 is positively correlated with the disease severity and bad prognostic outcomes in patients with COVID-19 (coronavirus disease 2019) 16. Consequently, these data revealed that IL-27 may involve in the pathogenesis of a range of pulmonary inflammatory diseases.

At present, the function of IL-27 in CAP patients remains unclear. The associations of serum IL-27 with the severity and prognosis were obscure among CAP patients. Therefore, it is reasonable to hypothesis that IL-27 may exert central influence in the pathophysiology progression of CAP. In order to explore the function of IL-27 in CAP patients, a prospective cohort study was conducted based on hospital population. The associations of serum IL-27 and the severity scores and prognostic outcomes were evaluated among CAP patients through this prospective cohort study.

## Methods

### Subjects

In total, 239 patients diagnosed with CAP that were admitted to the Department of Respiratory and Critical Care Medicine of the Second Affiliated Hospital of Anhui Medical University, Hefei City, Anhui Province between September, 2020 and April, 2021 were enrolled. Enrolled individuals were those meeting CAP clinical Practice Guidelines 17, 18. Patients were excluded if they were under 18 years of age, had suffered from other infectious diseases within the past 3 months, had undergone chemotherapy or radiotherapy to treat solid or hematological tumors, had received an organ transplant, were undergoing glucocorticoid treatment, presented with agranulocytosis or immunodeficiencies, or had been treated with immunosuppressive agents or cytokine antagonists within the last 6 months. An equal number of healthy control patients were additionally recruited from physical examination center in the Second Affiliated Hospital. Fasting blood samples were collected from CAP patients on admission prior to the initiation of antimicrobial treatment. Additionally, clinical characteristics and demographic information were extracted from the electronic patient record system. Patients' outcomes including duration of hospitalization, ICU admission, vasoactive agent use, mechanical ventilator and death were all tracked. CAP severity scores (PSI, CURXO, PSI, CURB-65, CRB-65, SMART-COP and APACHE Ⅱ) were assessed. All patients have provided informed consent in this research. The Research Ethics Committee of Second Affiliated Hospital of Anhui Medical University approved this study.

### Enzyme-linked immunosorbent assay (ELISA)

Fasting blood samples were obtained from healthy control individuals and CAP patients prior to the initiation of antimicrobial treatment. Fasting blood samples were centrifuged and serum samples were collected and subsequently stored at -87℃ refrigerator. Commercial ELISA kits for IL-27 (CSB-E08464h) and MIP-2 (CSB-E07420h) were obtained from Cusabio (Wuhan, China), while TNF-α ELISA kits (JYM0110Hu) were purchased from Wuhan Colorful Gene Biological Technology Co., Ltd. All ELISA kits were used based on provided directions 19-21.

### Statistical analysis

SPSS 20.0 was used for all statistical analysis. Data are expressed as means ± SEM or medians with interquartile ranges. The differences of clinical characteristics and demographic information were assessed with ANOVAs, Mann-Whitney U-tests, or Chi-squared tests among different groups. The correlations of serum IL-27 and clinical physiologic characters were analyzed using Spearman and Pearson linear analysis. Additionally, the associations between serum IL-27 with CAP severity scores and prognosis outcomes were estimated through linear and logistic regression analysis.* P* < 0.05 was the significance threshold.

## Results

### Demographic information and clinical characteristics

There were similar distributions of age, sex, body mass index (BMI), systolic blood pressure and diastolic blood pressure between CAP patients and control cases (Table 1). CAP patients exhibited higher rates of comorbidities including bronchitis, coronary heart disease, diabetes mellitus, hypertension, cerebral infarction, and other illnesses as compared to healthy controls. The average duration of hospitalization was 10.0 days among CAP patients. 70 (29.3%) patients suffered from ICU admission, 66 (27.6%) cases experienced mechanical ventilation, 34 (14.2%) patients underwent vasoactive agent use and 22 (9.2%) cases were dead during hospitalization. The severity of CAP was determined through CAP score systems, such as CURB-65, CRB-65, PSI, SMART-COP and APACHE Ⅱ (Table 1).

### Comparison of serum IL-27 levels in CAP patients and healthy controls

**Figure 1:**
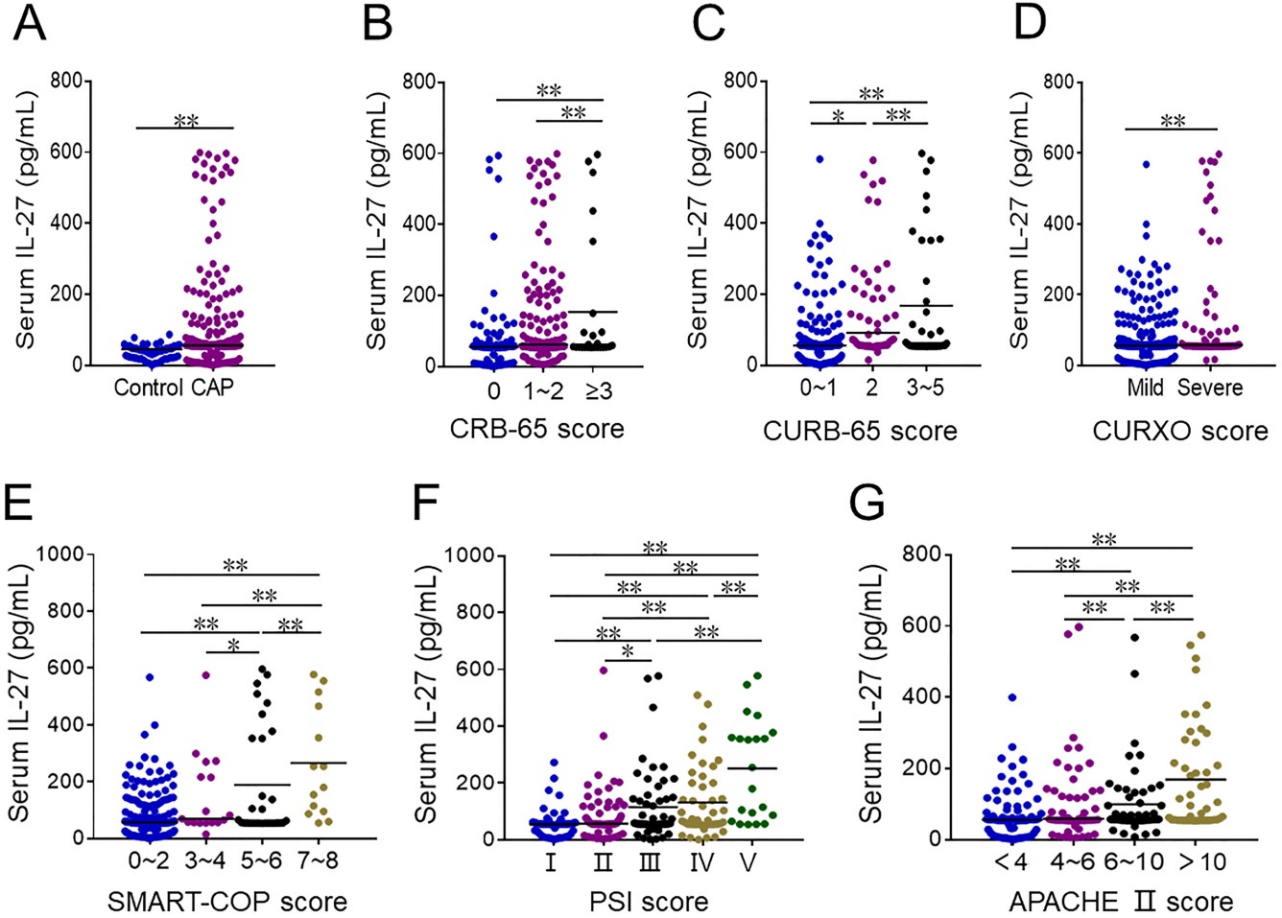
** The levels of serum IL-27 in CAP patients and healthy volunteers.** (A**-**G) Serum IL-27 was determined using ELISA in CAP patients and control subjects. (A) The levels of serum IL-27 in CAP patients and control cases. (B) The levels of serum IL-27 in patients with different CRB-65 scores. (C) The levels of serum IL-27 in patients with different CURB-65 scores. (D) The levels of serum IL-27 in patients with different CURXO scores. (E) The levels of serum IL-27 in patients with different SMART-COP score. (F) The levels of serum IL-27 in patients with different PSI scores. (G) The levels of serum IL-27 in patients with different APACHE Ⅱ scores. All data were expressed as mean ± SEM. *P < 0.05, **P < 0.01.

CAP patients exhibited significantly higher serum IL-27 levels as compared to healthy controls (Figure 1A). Among CAP patients, these levels were higher among individuals with a CRB-65 score ≥ 3 as compared to patients with scores of 0 or 1-2 (Figure 1B). Similarly, serum IL-27 levels rose with increasing CURB-65 scores (Figure 1C). Serum IL-27 levels were elevated in patients with severe disease, as established based upon CURXO scores, relative to those with mild disease (Figure 1D). With respect to SMART-COP grades, patients with scores of 3-4 or 5-6 exhibited higher serum IL-27 relative to patients with scores of 0-1, and these levels were highest in patients with a score of 7-8 (Figure 1E). IL-27 levels also were risen gradually with increasing PSI score (Figure 1F), and were lower for patients with an APACHE II score of < 4 compared to patients with scores of 4-6 or 6-10, while IL-27 levels were highest in CAP patients with a score of > 10 (Figure 1G).

### Correlations between serum IL-27 levels and clinicopathological characteristics in CAP patients

Correlations between serum IL-27 levels and routine blood indices were assessed in CAP patients, highlighting positive correlations between serum IL-27 with white blood cell (WBC) (*r=*0.332,* P<*0.001) and neutrophil (*r=*0.394,* P<*0.001) counts. Moreover, IL-27 level was negatively correlated with eosinophil *(r=-*0.262*, P <*0.001) and lymphocyte (*r=-*0.269,* P<*0.001) counts among CAP patients, whereas there was no obvious correlation of serum IL-27 with basophil or monocyte counts in CAP patients. With respect to indicators of renal, hepatic, and myocardial function, serum IL-27 levels were negatively correlated with uric acid levels (*r=-*0.215*, P<*0.001), but positively correlated with aspartate aminotransferase (AST) (*r=*0.332*, P=*0.022), alanine aminotransferase (ALT) (*r=*0.149*, P=*0.011), and cardiac troponin I (cTnI) (*r=*0.198, *P=*0.014) levels. Analyses of the relationship between serum IL-27 and coagulation function indicated a positive correlation between serum IL-27 and fibrinogen (FIB) (*r=*0.373*, P<*0.001), D-Dimer (*r=*0.339*, P<*0.001), and B-type natriuretic peptide (BNP) (r=0.210, *P*=0.009) among CAP patients. Lastly, the relationships between serum IL-27 and inflammatory cytokine were accessed among CAP patients, revealing that serum IL-27 was positively correlated with procalcitonin (PCT), macrophage inflammatory protein-2 (MIP-2), interleukin-6 (IL-6), c-reactive protein (CRP) in CAP patients (Table 2).

### The association of serum IL-27 with the severity of CAP

The associations of serum IL-27 and the severity scores were evaluated through linear and logistic regression analyses among CAP patients. Univariate linear and logistic regression analyses found strong positive correlations between serum IL-27 levels on admission with CRB-65 (β=0.303; 95% CI: 0.002~0.652), CURB-65 (β=0.325; 95% CI: 0.123~0.784), SMART-COP (β=0.306; 95% CI: 0.021~0.874), PSI (β=0.277; 95% CI: 0.048~0.127), APACHE Ⅱ (β=0.197; 95% CI: 0.04~0.019) and CURXO (OR=1.114; 95% CI: 1.002~1.257) among CAP patients (Table 3). After adjusted for sex and age, multivariate linear and logistic regression analyses suggested that serum IL-27 level was positively associated with CRB-65 (β=0.217; 95% CI: 0.021~0.795), CURB-65 (β=0.237; 95% CI: 0.001~2.345), SMART-COP (β=0.236; 95% CI: 0.002~0.025), PSI (β=0.160; 95% CI: 0.017~0.084) and CURXO (OR=1.113; 95% CI: 1.001~1.316) among CAP patients (Table 3).

### Comparison of serum IL-27 levels in CAP patients with different prognosis

**Figure 2:**
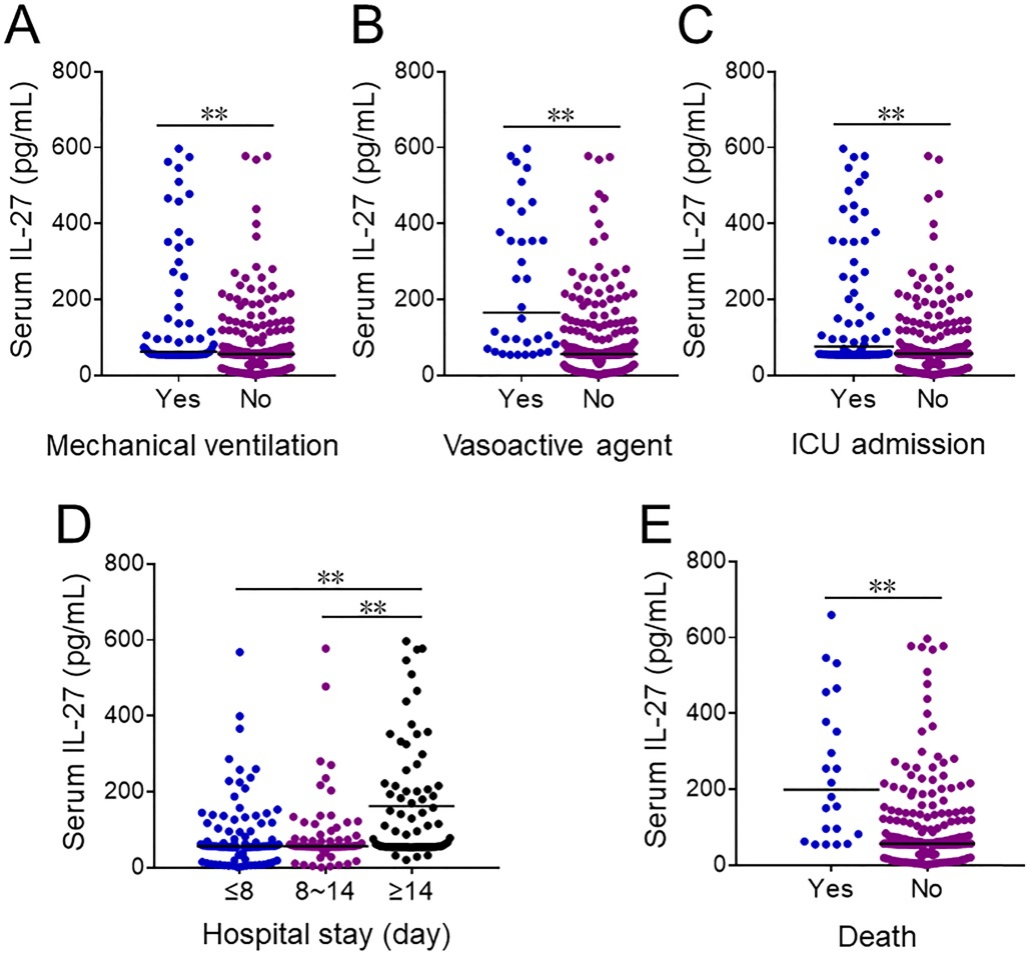
** The levels of serum IL-27 in CAP patients with different prognostic outcomes.** (A-E) The levels of serum IL-27 on admission were measured in CAP patients with different prognostic outcomes. (A) The levels of serum IL-27 in CAP patients with mechanical ventilation. (B) The levels of serum IL-27 in CAP patients with vasoactive agents. (C) The level of serum IL-27 in CAP patients with ICU admission. (D) The levels of serum IL-27 in CAP patients with different hospital stays. (E) The levels of serum IL-27 in dead cases and survived patients. All data were expressed as mean ± SEM. **P < 0.01.

Serum IL-27 levels were further compared in CAP patients with different prognostic outcomes. At the early stage of hospitalization, serum IL-27 level was upregulated in CAP cases with mechanical ventilation, vasoactive agents usage and ICU admission (Figure 2A-2C). Additionally, serum IL-27 level was compared among CAP patients with different length of stay on admission. Serum IL-27 level on admission was higher in ≥14 days than these in ≤8 and 8~14 days among CAP patients (Figure 2D). Moreover, serum IL-27 level on admission was increased in dead cased than those in survived patients (Figure 2E).

### The association of serum IL-27 with the prognosis in CAP patients

The associations of serum IL-27 and the prognostic outcomes were evaluated among CAP patients. Univariate logistic regression analysis identified positive associations between serum IL-27 and vasoactive agent usage (OR=1.123; 95% CI: 1.011~1.456), ICU admission (OR=1.113; 95% CI: 1.002~1.335), mechanical ventilator (OR=1.103; 95% CI: 1.001~1.305), hospitalization stays ≥14 days (OR=1.115; 95% CI: 1.011~1.438) and death (OR=1.035; 95% CI: 1.006~1.087) among CAP patients (Table 4). To eliminate potential confounding variables including sex and age, a multivariate logistic regression analysis was performed and then found that serum higher IL-27 on admission increased the risk of vasoactive agent usage (OR=1.112; 95% CI: 1.003~1.326) and hospitalization stays ≥14 days (OR=1.126; 95% CI: 1.003~1.462) in CAP patients (Table 4). Moreover, all CAP patients were divided into different grades in accord with CAP severity scores, including CURB-65, CRB-65, CURXO, SMART-COP and PSI. Based on the level of serum IL-27, the CAP patients in the one grade were divided into two groups, Higher IL-27 and Lower IL-27 groups. Then, the associations of serum IL-27 and the prognosis were evaluated in CAP patients with same severity. As shown in Table Supplemental 1, our results suggested that serum higher IL-27 on admission increased the risks of ICU admission, mechanical ventilation, vasoactive agent usage, death and hospital stays among CAP patients during hospitalization.

## Discussion

This study primarily evaluated the correlations of serum level of IL-27 with severity and prognosis in CAP patients using a prospective cohort study. This study mainly revealed that: (1) Serum IL-27 on admission was elevated in CAP patients; (2) Serum IL-27 on admission was gradually risen in parallel with CAP severity scores among CAP patients; (3) Serum IL-27 on admission was positively correlated with CAP severity scores in CAP patients; (4) Serum higher IL-27 on admission elevated the risk of vasoactive agent usage and longer hospital stays among CAP patients during hospitalization.

IL-27 is released by APCs in response to their TLR ligand- or pathogen-induced activation 8. IL-27 can, in turn, induce the secretion of a range of inflammatory cytokines from mast cells, monocytes and keratinocytes 9. In prior work, IL-27 has been shown to play a range of roles as a suppressor or enhancer of inflammatory activity, and it also exerts an immunomodulatory role in regulating Th 1 cells development 22. Moreover, there is growing evidence for the role of IL-27 as a contributor to a range of pulmonary diseases including ARDS, COPD, tuberculosis, asthma, acute lung injury and influenza 11-13. A study conducted *in vivo* suggested that IL-27 production can aggravate bleomycin-induced pulmonary fibrosis in mice 23. The functions of IL-27 in CAP patients are a clinical context, however, remains to be defined. To that end, this analysis was formulated to analyze serum IL-27 levels in CAP patients, revealing them to be significantly elevated as compared to levels in healthy volunteers. These IL-27 levels were gradually risen in line with CAP disease severity, and logistic regression analyses confirmed that higher serum IL-27 concentrations were associated with CAP severity scores, underscoring the close link between this cytokine and the progression of this infectious disease.

Several studies conducted by our group have highlighted a series of marked changes in routine blood indices and associated instances of multiple organ injury in patients with coronavirus disease 2019 (COVID-19) 24-27, spurring the present analysis of IL-27 levels and related clinical findings in CAP patients. Overall, these analyses revealed a negative correlation between serum IL-27 and lymphocyte levels in CAP patients, whereas IL-27 concentrations were positively correlated with the counts of WBC, neutrophils and monocytes, as well as key indicators of myocardial, renal, and hepatic function. Inflammation is a central driver of CAP incidence and progression 18, 28, 29, with a variety of inflammatory cytokines influencing the pathogenesis of microbe-induced CAP 30. Consistently, we herein detected a positive relationship between serum IL-27 levels with inflammatory cytokines and chemokines. Serum IL-27 may thus be a reliable biomarker indicative of disease severity in CAP patients.

Mortality rates among CAP patients remain persistently high 31, imposing a major medical and economic burden on affected individuals and societies throughout the globe 32. It is thus vital that approaches to reducing rates of CAP-associated mortality be identified. The timely diagnosis of CAP and the accurate evaluation of disease severity in infected-patients can help improve the odds of a positive outcome, making it essential that reliable biomarkers of poor CAP patient prognosis be clearly defined. One prior analysis identified a link between inflammatory parameters and prognosis in COVID-19 patients 33. Other researches have demonstrated that the levels of inflammation are increased in COVID-19 patients and inflammation repression attenuated the progression of COVID-19 34, 35. Work from our team has also previously clarified a negative correlation between the level of serum inflammatory cytokine S100A12 and poor prognostic outcomes in CAP patient 36. Herein, we assessed the link between serum IL-27 levels and prognosis in a CAP patient population, revealing a positive correlation between IL-27 level and both vasoactive agent utilization and longer duration of hospitalization among CAP patients with or without adjustment for patients' age and sex. Not only that, serum higher IL-27 also elevated the risks of poor prognosis in CAP patients with the same disease severity. As such, serum IL-27 levels may be a valuable prognostic biomarker for use in evaluating CAP patients.

These results have several key implications. Importantly, they offer direct evidence supporting a positive association between serum IL-27 concentrations with disease severity and adverse prognosis in CAP patients. However, there are multiple limitations to this study. Firstly, this was a single-center analysis of a relatively small population, and additional large-scale multicenter studies will thus be important to validate these findings. Secondly, as this was a prospective cohort study of a hospitalized patient population, the mechanisms whereby IL-27 upregulation was linked to CAP progression were not assessed. Further *in vitro* and *in vivo* research will thus be vital to better clarify this relationship. Thirdly, Il-27 levels were only analyzed in patients' serum and not in lung tissues or bronchoalveolar lavage fluids. Finally, the causative pathogens associated with CAP incidence in these patients were not identified.

## Conclusion

To summarize, this study primarily analyzed that relationship of serum IL-27 with the severity and prognosis among CAP patients through a prospective cohort study. These findings suggested that serum IL-27 is risen in CAP patients. Serum IL-27 on admission is gradually increased in parallel with the severity among CAP patients. In addition, serum higher IL-27 on admission is correlated with the severity and poor prognosis among CAP patients, indicating that IL-27 may take part in the pathophysiological process of CAP and be served as a potential biomarker for diagnose and prognose in CAP patients.

## Supplementary Material

Supplementary table.

## Figures and Tables

**Table 1:** Demographic characteristics of participators at baseline.

Variables	CAP (n=239)	Control (n=239)	*P*
Age (years)	64.0 (51.0, 75.0)	63.0 (52.0, 74.0)	0.351
Male, n (%)	143 (59.8)	155 (64.9)	0.256
BMI	22.1 (19.5, 24.8)	21.5 (19.0, 24.3)	0.089
Systolic pressure (mmHg)	126.0 (110.0, 141.0)	118.6 (103.5, 130.5)	0.125
Diastolic pressure (mmHg)	75.0 (67.0, 83.0)	71.0 (63.2, 80.5)	0.097
Comorbidities			
Hypertension, n (%)	64 (26.8)	21 (8.79)	<0.001
Diabetes mellitus, n (%)	22 (9.2)	6 (2.51)	0.002
Cerebral Infarction, n (%)	20 (8.4)	0	<0.001
Coronary heart disease, n (%)	11 (4.6)	0	0.001
Bronchitis, n (%)	19 (7.9)	0	<0.001
Other diseases, n (%)	78 (32.6)	11 (4.60)	<0.001
Hospital stays (day)	10.0 (7.0, 17.0)	N.A.	N.A.
ICU admission, n (%)	70 (29.3)	N.A.	N.A.
Mechanical ventilation, n (%)	66 (27.6)	N.A.	N.A.
Vasoactive agent, n (%)	34 (14.2)	N.A.	N.A.
Death, n (%)	22 (9.2)	N.A.	N.A.
CURB-65	1.0 (0, 2.0)	N.A.	N.A.
CRB-65	1.0 (0, 2.0)	N.A.	N.A.
PSI	72.0 (53.0, 97.0)	N.A.	N.A.
CURXO [Severe, n (%)]	66 (27.6)	N.A.	N.A.
SMART-COP	1.0 (0, 3.0)	N.A.	N.A.
APACHE Ⅱ	6.0 (4.0, 10.0)	N.A.	N.A.

**Table 2:** Associations between serum IL-27 and clinical characteristics in CAP patients.

Variables	WBC	Neutrophil	Lymphocyte	Monocytes	Eosinophil	Basophil
***r***	0.332	0.394	-0.269	0.079	-0.262	-0.067
***P***	<0.001	<0.001	<0.001	0.115	<0.001	0.152
Variables	Uric acid	Urea nitrogen	Creatinine	ALT	AST	CK
***r***	-0.215	0.075	0.086	0.149	0.332	0.098
***P***	<0.001	0.126	0.094	0.011	0.022	0.110
Variables	CKMB	cTnI	D-Dimer	PT	BNP	PLT
***r***	0.030	0.198	0.339	0.105	0.210	0.085
***P***	0.355	0.014	<0.001	0.056	0.009	0.097
Variables	PCT	FIB	TNF-α	MIP-2	IL-6	CRP
***r***	0.391	0.373	0.556	0.456	0.597	0.482
***P***	<0.001	<0.001	<0.001	<0.001	<0.001	<0.001

**Table 3:** Associations between serum IL-27 and CAP severity scores in CAP patients.

	Univariable	*P*	Multivariable*	*P*
	β (95% CI)		β (95% CI)	
CRB-65	0.303 (0.002, 0.652)	<0.001	0.217 (0.021, 0.795)	<0.001
CURB-65	0.325 (0.123, 0.784)	<0.001	0.237 (0.001, 2.354)	<0.001
SMART-COP	0.306 (0.021, 0.874)	<0.001	0.236 (0.002, 0.025)	<0.001
PSI	0.277 (0.048, 0.127)	<0.001	0.160 (0.017, 0.084)	0.003
APACHE Ⅱ	0.197 (0.004, 0.019)	0.002	0.116 (0.001, 0.014)	0.055
	OR (95% CI)		OR (95% CI)	
CURXO	1.114 (1.002, 1.257)	0.001	1.113 (1.001, 1.316)	0.006

* Adjusted for age and sex.

**Table 4:** Association between serum IL-27 and prognosis in CAP patients.

		Univariable (95% CI)	*P*	Multivariable (95% CI) *	*P*
ICU admission		1.113 (1.002, 1.335)	0.011	1.002 (1.000, 1.004)	0.092
Mechanical ventilation		1.103 (1.001, 1.305)	0.010	1.002 (1.000, 1.011)	0.077
Vasoactive agent		1.123 (1.011, 1.456)	0.004	1.112 (1.003, 1.326)	0.022
Death		1.035 (1.006, 1.087	0.045	1.002 (0.999, 1.005)	0.145
Hospital stays					
	≤8	1	——	1	——
	8~14	1.000 (0.997, 1.004)	0.832	1.001 (0.996, 1.009)	0.835
	≥14	1.115 (1.011, 1.438)	0.005	1.126 (1.003, 1.462)	0.037

* Adjusted for age and sex.

## Abbreviations

CAP: Community-acquired pneumonia
Interleukin-27: IL-27
ELISA: Enzyme linked immunosorbent assay
WBC: White blood cell
CRP: C-reactive protein
ALT: Alanine aminotransferase
AST: Aspartate aminotransferase
cTnI: Cardiac troponin I
FIB: fibrinogen
BNP: B-type natriuretic peptide
PCT: procalcitonin
TNF-α: tumor necrosis factor
MIP-2: Macrophage inflammatory protein-2
IL-6: Interleukin-6
