# Supplementary material for: Serum IL-27 predicts the severity and prognosis in patients with community-acquired pneumonia: a prospective cohort study
Source: Int J Med Sci. 2022 Jan 1;19(1):74–81. doi: 10.7150/ijms.67028 (PMC8692123; doi:10.7150/ijms.67028)
Supplement: Supplementary file 1 — Supplementary table. [file ijmsv19p0074s1.pdf]

Table Supplemental 1. The associations of serum IL-27 with the prognosis in CAP patients with same severity

CAP score system	Total, N	Higher IL-27, N	ICU Admission, N (%)	Mechanical ventilation, N (%)	Vasoactive agent, N (%)	Death, N (%)	Hospital stays, N (%)
CURB-65							
0~1	148	64	4 (6.2)	4 (6.2)	3 (4.7)	2 (3.1)	12 (18.8)
2	50	35	13 (37.1)	13 (37.1)	5 (14.3)	3 (8.6)	14 (40.0)
3~5	37	19	15 (78.9)	15 (78.9)	12 (63.2)	8 (42.1)	13 (68.4)
CRB-65							
0	84	71	1 (2.8)	1 (2.8)	1 (2.8)	1 (2.8)	4 (11.1)
1~2	126	118	21 (30.0)	22 (31.4)	12 (17.1)	7 (10.0)	26 (41.1)
⩾3	25	15	10 (83.3)	9 (75.0)	7 (58.3)	5 (41.7)	9 (75.0)
CURXO							
Mild	62	86	9 (10.5)	10 (11.6)	5 (5.8)	2 (2.3)	19 (22.1)
Severe	173	32	23 (71.9)	22 (68.8)	15 (46.9)	11 (34.4)	17 (19.5)
SMART-COP							
0~2	174	85	8 (9.4)	8 (9.4)	3 (3.5)	1 (1.2)	18 (21.2)
3~4	17	11	7 (63.6)	7 (63.6)	3 (27.3)	3 (27.3)	4 (36.4)
5~6	30	15	11 (73.3)	11 (73.3)	8 (53.3)	5 (33.3)	10 (66.7)
7~8	14	7	6 (85.7)	6 (85.7)	6 (85.7)	4 (57.1)	7 (100.0)
PSI							
I	54	20	2 (10.0)	2 (10.0)	1 (5.0)	2 (10.0)	4 (20.0)
II	61	30	1 (3.3)	1 (3.3)	1 (3.3)	0	7 (27.3)
III	55	28	4 (14.3)	5 (17.9)	2 (7.1)	3 (10.7)	8 (28.6)
IV	46	28	11 (39.3)	12 (42.9)	6 (21.4)	2 (7.1)	10 (35.7)
